# Wolfram-like Syndrome: Shedding Light on a Variant of Wolfram Syndrome

**DOI:** 10.1016/j.aed.2025.06.008

**Published:** 2025-07-03

**Authors:** Miranda Matzer, Ricardo A. Caravantes, Karoline Marie Schieber López, Marta González-Salaices, Miriam Eimil, Beatriz Oyanguren, Patricia Gomez, Pilar Alcántara, Cristina Prieto, Sarai Urtiaga, Sofia Portela, Ignacio Casanova

**Affiliations:** 1 Department of Medical Research, Universidad Francisco Marroquin, Guatemala City, Guatemala; 2 Department of Opthalmology, Universidad de San Carlos de Guatemala, Guatemala City, Guatemala; 3 Department of Medical Research, Hospital Universitario Torrejon, Madrid, Spain

**Keywords:** diabetes mellitus, optic atrophy, *WFS1* gene, Wolfram-like syndrome

## Abstract

**Background:**

Wolfram-like syndrome is an autosomal dominant disorder related to classical autosomal recessive Wolfram syndrome. It is characterized by diabetes mellitus, optic atrophy, and sensorineural hearing loss, but typically presents with milder or incomplete features. These atypical and late-onset forms pose a diagnostic challenge.

**Case Report:**

A 58-year-old male presented with progressive bilateral visual loss, hearing loss, and a history of diabetes mellitus. Optical coherence tomography revealed bilateral optic atrophy, and genetic testing identified a heterozygous pathogenic variant, NM_006005.3:c.409_424dup (p.Val137Glyfs∗110), in the *WFS1* gene, confirming the diagnosis of Wolfram-like syndrome.

**Discussion:**

This case highlights the variability in clinical presentation of Wolfram-like syndrome, including unusually late symptom onset. It also emphasizes the role of genetic modifiers and the importance of distinguishing this condition from classical Wolfram syndrome, particularly in adult-onset cases.

**Conclusion:**

Early recognition of Wolfram-like syndrome in atypical presentations is critical for appropriate management and patient counseling. The objective of this report is to describe a case of Wolfram-like syndrome with unusually late onset, underline its distinguishing features, and raise awareness of this under-recognized disorder.

**Highlights**

• Wolfram-like syndrome presents with milder features than classical Wolfram syndrome
• Genetic testing revealed a heterozygous pathogenic mutation in the *WFS1* gene
• Early diagnosis is essential for managing progressive symptoms
• Multidisciplinary care improves patient outcomes in progressive syndromes

**Clinical Relevance**

This case underscores the importance of considering Wolfram-like syndrome in patients with diabetes, optic atrophy, and hearing loss. Early genetic testing and a multidisciplinary team approach are crucial for managing the progressive nature of the disease and improving patient outcomes.

## Introduction

Wolfram syndrome (WFS) is a rare, neurodegenerative disorder with a prevalence of approximately 1 in 500 000 individuals worldwide^1^. It is primarily characterized by early-onset, nonimmune, insulin-dependent diabetes mellitus and progressive optic atrophy, typically manifesting within the first decade of life. Additional features, including diabetes insipidus and sensorineural deafness, are observed in approximately 70% of cases. Beyond these hallmark features, WFS may also present with psychiatric, neurologic, endocrine, cardiac, urinary tract, and reproductive system abnormalities, further delineating its multisystemic nature^2^^,^^3^.

WFS is classified into 2 types (WFS1 and CISD2 gene mutations), with WFS1 being the most common and typically linked to autosomal recessive mutations^4^. The WFS1 gene on chromosome 4p16 encodes wolframin, a transmembrane protein essential for regulating endoplasmic reticulum calcium homeostasis and apoptosis; disruptions in these processes drive the syndrome's pathophysiology^3^. More than 200 different variations in WFS1 have been described, with different effects on gene and protein expression, which complicates the establishment of clear genotype–phenotype correlations^5^. Moreover, although WFS is classically considered an autosomal recessive disease, recent advances in genetic testing have shown the absence of a second mutant allele in at least 8% to 9% of patients. This may be attributed to the inability to detect a second mutation, the presence of dominant mutations, or mutations in another gene. In such cases, Wolfram-like syndrome (WFLS) has been described as a spectrum of the classical WFS with milder clinical manifestations^5^^,^^6^.

Despite its low prevalence, WFS and WFLS are progressive diseases with no specific treatment due to their multisystem involvement and variability in severity^3^^,^^4^. However, early identification of the syndrome or its constituent symptoms can facilitate supportive care and regular clinical follow-up, which are crucial for improving quality of life in affected individuals^2^.

We present a case of WFLS characterized by progressive, severe visual loss, accompanied by only mild additional manifestations. This atypical, late-onset presentation initially made it difficult to suspect WFS1 mutations as the underlying cause. Our objective is to emphasize the importance of genetic testing in complex cases, and to contribute to the understanding of WFLS as a milder condition associated with heterozygous WFS1 mutations, in contrast to the classical homozygous form observed in WFS.

## Case Report

The patient is a 58-year-old male who presented to the neurology department with progressive bilateral visual acuity loss, which began approximately 2 months earlier. He had a known history of diabetes mellitus, diagnosed 2 years prior, managed with metformin 850 mg, half a tablet every 12 hours. He had also experienced long-standing hearing loss and tinnitus, for which he had not sought medical attention until 3 months before the onset of visual symptoms. Audiometry revealed bilateral high-frequency hearing loss with thresholds up to 60 dB.

The patient sought help at a private ophthalmology clinic, where he was diagnosed and surgically treated for cataracts 1 month before seeking care with neurology. However, his visual loss continued to progress rapidly after the surgery. He had never experienced significant visual problems before.

His family history is significant for Parkinson disease in his mother and prostate cancer in his father. No other medical conditions were reported in his parents, particularly no hearing loss, visual impairment, or diabetes. The patient has 1 brother, 3 years older, with no known medical history.

On physical examination, the patient’s vital signs were stable, and the neurological exam was unremarkable, with no focal deficits observed. The ophthalmological examination revealed a visual acuity of 0.05 in the right eye (OD) and 0.25 in the left eye (OS). No relative afferent pupillary defect was noted. Dyschromatopsia was not formally tested; however, the patient reported altered color perception in both eyes, with the exception of blue. Optical coherence tomography revealed severe, bilateral thinning of the macular ganglion cell layer (GCL), with average thickness values of 64 μm in the OD and 63 μm in the OS, significantly below the normal range (80–100 μm). Minimum GCL thickness was also reduced (53–57 μm), indicating diffuse ganglion cell loss. Retinal nerve fiber layer (RNFL) analysis showed mild average thinning in the inferior quadrant of both eyes (61 μm OD, 60 μm OS), with increased cupping and cup volume suggestive of chronic axonal damage. RNFL asymmetry (66%) pointed to a nonuniform pathological process.

The perifoveal macular map showed preserved central thickness with surrounding midperipheral thinning, consistent with inner retinal layer loss. Sectoral GCL analysis demonstrated bilateral, diffuse thinning, with most regions falling below the first percentile and with slightly more pronounced thinning in the left eye. Altogether, these findings show a pattern of bilateral and symmetrical ganglion cell thinning, with greater involvement of the papillomacular fibers, which is characteristic of hereditary optic atrophy. These findings correlate with the patient’s visual field defects—central scotoma in the right eye and centrotemporal scotoma in the left—and further support the diagnosis.

Routine blood tests and cerebrospinal fluid analysis were within normal limits. Islet autoantibody, C-peptide and HbA1c were not obtained at the time of presentation. Magnetic resonance imaging showed no structural brain abnormalities, optic nerve involvement, or posterior fossa lesions. Of note, this apparent discrepancy is explained by the higher sensitivity of optical coherence tomography, which can detect microstructural axonal loss in the RNFL before optic nerve atrophy becomes radiologically apparent on conventional magnetic resonance imaging^7^. Angiotomography of the circle of Willis and supra-aortic trunks also revealed no morphological abnormalities.

Given the clinical presentation, a genetic etiology was considered. Empirical treatment with idebenone (900 mg/d) was initiated. Testing for Leber Hereditary Optic Neuropathy was negative; however, next-generation sequencing identified a heterozygous pathogenic variant, NM_006005.3:c.409_424dup (p.Val137Glyfs∗110), in the WFS1 gene, confirming the diagnosis of WFLS. Both of the patient’s first-degree relatives are deceased and were not available for genetic testing; therefore, de novo status could not be determined. The patient was referred for genetic counseling and his family members were informed of the findings. Given the negative Leber Hereditary Optic Neuropathy result and the fact that mitochondrial dysfunction is not the primary pathophysiological mechanism in WFLS, idebenone was discontinued. Regarding his diabetes mellitus, metformin was replaced with empagliflozin due to its more favorable oxidative stress profile and its lower risk of lactic acidosis and hypoglycemia, both of which could exacerbate optic neuropathy. His visual acuity continued to deteriorate during subsequent visits, with worsening central scotoma and reduced peripheral vision. Fortunately, the patient reported stability in his neurological status, with no significant progression in other organ systems. Ongoing care focused on supportive measures and multidisciplinary management. The patient received occupational therapy to adapt to his visual impairment, psychiatric and psychological support, and bilateral hearing aids, as well as regular follow-up in neurology and ophthalmology clinics.

## Discussion

This case highlights the clinical and genetic features of WFLS, a recently described neurodegenerative disorder related to WFS but inherited in an autosomal dominant pattern^6^^,^^7^. The diagnosis was confirmed by identifying a heterozygous pathogenic variant, NM_006005.3:c.409_424dup (p.Val137Glyfs∗110), in the WFS1 gene in the context of the patient’s optic neuropathy, diabetes mellitus, and hearing loss. The variant was classified as pathogenic based on ACMG/AMP guidelines, using the following evidence codes: PVS1 (indicating a loss-of-function mutation in a gene where this is a known disease mechanism), PM2 (the variant is absent from population databases), and PP3 (computational tools predict the variant is harmful)^8^^,^^9^.

The clinical phenotype in this case is most consistent with haploinsufficiency as the underlying mechanism. A single loss-of-function allele in WFS1 can lead to WFLS, as reduced protein levels are insufficient to maintain normal cellular function. There is little evidence to suggest that the variant NM_006005.3:c.409_424dup (p.Val137Glyfs110) acts through a dominant-negative mechanism. Since this is a frameshift mutation introducing a premature stop codon early in the transcript, it is highly likely to undergo nonsense-mediated mRNA decay, preventing the production of a truncated or interfering protein. This interpretation is supported by recent genotype–phenotype correlation studies, which describe haploinsufficiency as the predominant cause of autosomal dominant WFLS in loss-of-function variants^6^^,^^10^.

### Atypical Late-Onset Presentation

WFLS is generally associated with later and milder onset than classical WFS; however, it can still show considerable clinical variability. Symptom onset in this patient’s late 50s, with rapid clustering of features over a short timeframe, represents an unusually late and diagnostically challenging presentation of WFLS. According to a systematic review by de Muijnck et al, most individuals with WFLS develop symptoms in adolescence or early adulthood^6^. The NM_006005.3:c.409_424dup variant accounts for approximately 6% to 7% of reported WFS1 mutations^11^. Although no definitive genotype–phenotype correlation has been established in WFLS, frameshift and nonsense mutations tend to produce more severe presentations than missense variants; therefore, the presence of this frameshift mutation may explain the severity of our patient’s visual impairment^5^^,^^6^. Interestingly, the father of the index case described in a report by SAR Journal of Medical Case Reports carried the same variant yet remained asymptomatic, suggesting variable penetrance potentially influenced by modifier genes, epigenetic regulation, or environmental factors^12^.

### Importance of Early Diagnosis and Supportive Management

Disease progression in WFLS is difficult to anticipate due to its variable and unpredictable course, requiring regular monitoring in the absence of curative treatments ^3^^,^^13^. Early and accurate recognition is essential for guiding supportive care and optimizing long-term management. Although no treatment can halt disease progression, addressing complications such as diabetes and visual loss can significantly improve quality of life. In this patient, the progressive visual decline and hearing loss emphasize the need for multidisciplinary care involving neurology, ophthalmology, audiology, and endocrinology. While the prognosis for WFLS is generally better than that of classical WFS, severe manifestations, as exemplified by this patient’s rapid visual decline, demonstrate the potential for a poor outcome without comprehensive and coordinated care.

## Conclusion

This case illustrates the diagnostic complexity of WFS and WFLS, particularly in atypical, late-onset presentations. The identification of a heterozygous pathogenic variant in the WFS1 gene underscores the variability in phenotypic expression in these diseases. Regular follow-up and multidisciplinary care are essential for managing progressive complications and optimizing patient outcomes. Genetic testing plays a crucial role in confirming rare diagnoses, guiding management strategies, and informing family counseling.

## Patient Consent

Written informed consent was obtained from the patient before the inception of this case report, ensuring their voluntary participation.

## Disclosure

The authors have no conflicts of interest to disclose.
